# A Comprehensive Exploration of the Fatty Acids Profile, Cholesterol, and Tocopherols Levels in Liver from Laying Hens Fed Diets Containing Nonindustrial Hemp Seed

**DOI:** 10.1155/2024/8848436

**Published:** 2024-01-04

**Authors:** Youssef Rbah, Yassine Taaifi, Aymane Allay, Kamal Belhaj, Reda Melhaoui, Nadia Houmy, Abdessamad Ben Moumen, Embarek Azeroual, Mohamed Addi, Farid Mansouri, Hana Serghini-Caid, Ahmed Elamrani

**Affiliations:** ^1^Laboratory for Agricultural Productions Improvement, Biotechnology and Environment, Faculty of Sciences, University Mohammed First, BP-717, Oujda 60000, Morocco; ^2^Laboratory of Sustainable Agriculture Management, Higher School of Technology Sidi Bennour, University Chouaib Doukkali, Street Jabran Khalil Jabran BP 299-24000, El Jadida, Morocco; ^3^Agro-Food Technology and Quality Laboratory, Regional Center of Agricultural Research of Oujda National Institute of Agricultural Research, Ennasr Av, BP 415, Rabat 10090, Morocco; ^4^Royal Institute of Livestock Fouarat, Kenitra, Morocco; ^5^Laboratory LSAIP Higher School of Education and Training, Mohammed I University, BP-410, Oujda 60000, Morocco

## Abstract

This study investigates the impact of dietary nonindustrial Moroccan hemp seed (HS) on the fatty acid profile, cholesterol, and tocopherol levels, in the liver of 120 Lohmann brown laying hens aged 22 weeks during 12 weeks of treatment. The hens are randomly allocated into four treatment groups, each subdivided into six replicates with five birds in each replicate. The dietary treatments consist of 0% HS (control), 10% HS, 20% HS, and 30% HS. Results indicate a substantial increase (*p* < 0.01) in polyunsaturated fatty acids, including omega 3 (n-3) and omega 6 (n-6) types, with the inclusion of HS in the diet. The n-6/n-3 ratio is significantly reduced (*p* < 0.01), and there is a significant reduction (*p* < 0.01) in saturated fatty acids only for the 30% HS treatment, indicating a more favorable fatty acid composition. Cholesterol levels remain largely unaffected by HS inclusion, except for the 10% HS group, which shows a significant decrease (*p* < 0.05). Moreover, hepatic tocopherol levels are significantly elevated (*p* < 0.01) in subjects receiving the HS diet, with the 30% HS group exhibiting the highest tocopherol content. In summary, incorporating HS into the diet up to 30% appears to offer promising benefits for hepatic lipid composition, particularly in terms of n-3 polyunsaturated fatty acids, the n-6/n-3 ratio, and tocopherol levels, while having minimal impact on cholesterol levels.

## 1. Introduction

Eggs are an important human foodstuff, consumed in millions of tonnes every year. According to statistics, world production is estimated at 86.4 million tonnes in 2021 and is set to rise further due to the strong demand for animal proteins [1]. Hens' eggs constitute a great source of proteins and fats as well as an excellent and balanced source of essential amino acids, certain minerals (calcium, zinc, and iron), and vitamins both liposoluble and hydrosoluble [2–5]. Daily consumption of eggs provides almost all of the recommended amount of these nutrients [6].

Poultry farming in Morocco has undergone rapid growth in recent years, with a 44% surge in investment recorded between 2010 and 2019. Nevertheless, the sector's heavy reliance on imported poultry feed inputs remains a significant challenge. Hence, a key research focus has been to explore the feasibility of utilizing local ingredients that can lower feed costs while maintaining high nutritional standards for the hens. One promising alternative is the incorporation of fish-based products such as fish oils and meals produced within Morocco, as well as seaweed-based ingredients. Nevertheless, the high cost and the sustainability of the world's fish stocks remain a serious concern. In addition, some research has indicated their adverse impact on both hens' productive performance and the sensory attributes of the resultant eggs [7]. A further alternative would be to use oilseeds produced in Morocco, such as local nonindustrial cannabis seeds (named beldiya). Beldiya is considered a hashish terroir. The hashish production in Morocco relies on a specific cultivar named NLD, an abbreviation for narrow-leaf drug cannabis, often colloquially termed “sativa.” This particular cultivar, *Cannabis indica* ssp. indica var. mediterranean, may have emerged from the interbreeding of Asian NLD with narrow-leaf Cannabis varieties (NLH) from southern Europe. This hybridization could account for the cultivar's moderate delta-9-tetrahydrocannabinol (THC) content, ranging from 2% to 5%, and its comparatively elevated cannabidiol (CBD) levels [8].

In previous studies conducted in our laboratory [9, 10], the variability of oil, tocopherol, phenol, and fatty acid content across various nonindustrially produced Moroccan hemp seed (HS) varieties was investigated. The fatty acid composition predominantly consisted of unsaturated fatty acids (87.3–88.96%), with linoleic acid (LA) being the dominant form, accounting for approximately 56% of total fatty acids, while *α*-linolenic acid (ALA) levels ranged from 14 to 17%. The LA (omega-6) to ALA (omega-3) ratio was found to be approximately 3.4. Furthermore, our findings [10, 11] demonstrate that HS collected from different regions of the Rif region exhibited significant concentrations of phenolic compounds and gamma tocopherols, along with potent antioxidant activity that could provide protection to PUFA, particularly very long-chain fatty acids (VLCs), against oxidation.

Until now, most investigations on the impact of incorporating hemp seed (HS) or hemp oil in poultry diets have primarily focused on productive performance and egg/meat nutritional quality [12–14].

Numerous research studies have been conducted to assess the influence of industrial hemp seeds on the modification of lipid profiles in the hepatic tissue of chickens [15–17]. Yang et al. [18] revealed that dietary alterations can induce variations in lipogenesis and lipid storage in the livers of chickens. By combining elevated quantities of hemp seeds with flaxseeds, the hepatic vitamin E content was augmented [17]. Elkin et al. [19] established that the addition of DHA-rich microalgae to the diets of White Leghorn hens mitigated substantial liver enlargement, induced changes in the ovarian follicle hierarchy, reduced the circulation of triglycerides, as well as suppressed hepatic expression of critical genes linked to the biosynthesis of triglycerides.

In a previous study [20], it was documented that the utilization of nonindustrial Moroccan hemp seeds in the diets of Lohmann Brown laying hens resulted in a marked enhancement in nutritional egg quality, which was attributed to elevated levels of n-3 fatty acids and tocopherols. The objective of the current investigation was to explore the impact of integrating nonindustrial Moroccan hemp seeds from the Beldiya ecotype into the diets of laying hens on the hepatic fatty acid profile, tocopherols, and cholesterol content, while concurrently correlating these alterations with observed nutritional composition changes in eggs.

## 2. Materials and Methods

This experiment has been performed in the poultry building at the Royal Institute of Livestock, Kenitra, kingdom of Morocco.

### 2.1. Birds and Husbandry

In this investigation, a group of 120 Lohmann Brown laying hens, each aged 22 weeks, was procured from a licensed commercial poultry facility. These hens underwent an initial weight assessment and were subsequently assigned randomly to one of four distinct treatment groups, which encompassed a control group receiving no hemp seed supplementation. Each treatment group was subdivided into six replicates, each consisting of five birds, and a completely randomized design was employed. The dietary interventions involved the substitution of soybean-corn components in the control basal diet with varying levels of hemp seed, specifically 0% (control), 10% (HS 10), 20% (HS 20), and 30% (HS 30). The laying hens were accommodated in a designated animal housing facility equipped with automated drop belts and battery cages, with the following dimensions: 0.61 m long, 0.57 m wide, and 0.5 m high, adhering to rigorous sanitary and veterinary standards. Individual nipple drinkers were provided for each bird, and the metallic feeders were partitioned to prevent interreplicate feed consumption. Feeding occurred thrice daily, and *ad libitum* access to water was ensured. Environmental conditions were meticulously regulated, maintaining a controlled temperature range of 18–22°C, humidity levels at 55–60%, and a photoperiod of 16 hours of light followed by an 8-hour dark cycle. The experiment lasted 12 weeks; throughout the experimental duration, no instances of mortality were observed, and vaccination procedures were executed under the oversight of a licensed veterinarian.

### 2.2. Experimental Diets

The composition of the diets and ingredients used were described in our previous article (Table 1) [20]. As for the content of the hemp seeds used in this study, they are shown in (Table 2). The diets administered were carefully formulated to be isocaloric and isonitrogenous, meticulously tailored to meet the specific nutritional needs of laying hens [21], receiving a daily ration of 100 grams of feed [18]. The diets were administered on a daily basis and stored at a temperature of 4°C throughout the duration of the trial. The dietary formulations encompassed incremental quantities of HS (0%, 10%, 20%, and 30%), designated as control, HS-10, HS-20, and HS-30, respectively, incorporated into a primarily soybean-corn-based mixture. These diets were produced by a specialized poultry feed company called BENWAY.

### 2.3. Diet Analyses

In the research, an examination was conducted on the nutritional elements within the diet, encompassing moisture, ash, crude protein, crude fiber, calcium, total phosphorus, and ether extract contents, as per the methodology outlined by [22]. The apparent metabolizable energy (AME) of the key dietary components was assessed following the European reference approach described by [23]. This method involves calculating the difference between energy intake and energy expenditure.

### 2.4. Sampling

At the end of the experiment, when layers had reached 36 weeks of age, the hens were transported to a slaughterhouse. Six hens from each treatment group (one hen from each replicate) were randomly selected and weighed. Prior to slaughter, the layers were subjected to a 12-hour feed withdrawal, then slaughtered according to the halal cutting method before being plucked and eviscerated. This method of slaughter involves first holding the hens' legs with the right foot and the wings with the left. The protocol begins with the recitation of Allah's name and the Tasmiyah (Bismillah Allahu Akbar). Then, using a very sharp knife, the neck of the chicken was cut down to the bone. The weight of the carcass was recorded prior to tissue sampling. The liver was expeditiously removed from the carcass, weighed, and rapidly cooled using liquid nitrogen and then preserved at a temperature of −20°C for future analyses, including fatty acid assessment. All relevant international and national regulations and ethical protocols governing the humane treatment and utilization of animals were meticulously adhered to throughout the study [24].

### 2.5. Liver Lipid Content and Cholesterol

The Folch technique was employed to extract total lipids from liver and diet samples. This process involved homogenization in a solution composed of chloroform and methanol (2 : 1, v/v), as initially outlined by the 2 : 1 (v/v) solution [25]. Following centrifugation, the organic phase was collected, and the resulting all-lipid extracts were retained for subsequent analyses. To determine the levels of cholesterol in the liver, the Liebermann–Burchard (LB) technique, as detailed by Sabir et al. [26], was employed. Specifically, 0.1 g of hepatic fat was diluted in 10 ml of chloroform, and 3 ml aliquots were extracted, followed by the addition of 2 ml of LB reagent (comprising 0.5 ml sulfuric acid dissolved in 10 ml of acetic anhydride). The final volume was adjusted to 7 ml with chloroform. In parallel, a standard cholesterol solution (1 mg/ml) was prepared, and 5 aliquots ranging from 0.5 ml to 2.5 ml were treated with 2 ml of LB reagent, with the final volume adjusted to 7 ml using chloroform. The solution was then incubated in darkness for 15 minutes, and the absorbance was measured at 640 nm using a UV spectrophotometer (Rayleigh UV1800, UV-Visible).

### 2.6. Fatty Acid Analyses

To analyze fats, the AOCS “Ce 1k-09” method was employed to transform fatty acids from diet and liver samples into fatty acid methyl esters (FAMEs) [27]. Subsequently, these fats were subjected to analysis via gas chromatography, coupled with a flame ionization detector (GC Agilent 6890, Agilent Technologies). The injection of FAMEs into the system was performed in a splitless mode, with 1 *μ*L of the sample injected. Separation of FAMEs took place in a BPX70 capillary column (60 m long, 0.32 mm internal diameter, and 0.25 *μ*m film thickness; SGE Europe). Helium gas served as the carrier gas at a flow rate of 1 mL per minute. Initially, the oven temperature was set at 50°C, subsequently increased to 170°C at a rate of 30°C per minute, and then raised by 4°C per minute until reaching 220°C. This temperature was maintained for 10 minutes. Identification of the fatty acids was accomplished by comparing them to a standard containing 37 FAMEs (Sigma-Aldrich, Supelco, Bellefonte PA, USA), and the results were reported as percentages.

### 2.7. Calculation of Health Lipid Indices

The health lipid indices profile of the enriched liver hens was determined by analyzing the proportions of specific fatty acids and their groups. This involved calculating various indices, including saturation indices (SI), desirable fatty acids (DFAs), atherogenic indices (AI), thrombogenic indices (TI), oxidative susceptibility (OS), the ratio of hypocholesterolemic and hypercholesterolemic fatty acids (hH), hypercholesterolaemic saturated fatty acids (HFSA), and nutritive value indices (NVI). The following relevant formulas were used to calculate each of these indices [28–30]:

(1) $$\begin{array}{c} \mathrm{SI}=\left[\frac{\left(C14:0+C16:0+C18:0\right)}{\left(\text{MUFA}+\text{PUFA}\right)}\right],\\ \mathrm{DFA}=\left[\left(C18:0+\text{UFA}\right)\right],\\ \mathrm{AI}=\left[\frac{\left(4\times C14:0+C16:0+C18:0\right)}{\left(\text{MUFA}+\text{PUFA}\right)}\right],\\ \mathrm{TI}=\left[\frac{\left(\Sigma \text{SFA}\right)}{\left(\left(0.5\times \Sigma \text{MUFA}\right)+\left(0.5\times n-6\text{PUFA}\right)+\left(3\times n-3\text{PUFA}\right)+\left(n-3:n-6\right)\right)}\right],\\ \mathrm{OS}=\left[\left(\text{MUFA}+\left(45\times C18:2\right)+\left(100\times C18:3\right)\right.\right.,\\ \frac{h}{H}=\left[\frac{\left(C18:1n-9+C18:2n-6+C20:4n-6+C18:3n-3+C20:5n-3+C22:6n-6\right)}{\left(C14:0+C16:0\right)}\right],\\ \mathrm{HSFA}=\left[\left(C14:0+C16:0\right)\right],\\ \mathrm{NVI}=\left[\frac{\left(C18:0+C18:1\right)}{C16:0}\right]. \end{array}$$

### 2.8. Tocopherols Analyses

To examine tocopherol compounds in liver fat, the methodology by Ben Moumen et al. [31]. We employed high-performance liquid chromatography with diode array detection (HPLC-DAD) for this purpose. Initially, the samples were dissolved in hexane and then separated using an Uptisphere 120 A NH2 silica column (250 mm × 4.6 mm, 5 *μ*m). The mobile phase used was a hexane/isopropanol mixture (99 : 1, v/v) maintained isocratically. The flow rate was set at 1 mL/min, and a UV detector was used to identify the separated compounds at wavelengths of 292, 296, and 298 nm. For quantification, we relied on external calibration, utilizing a commercially available tocopherol standard from Sigma-Aldrich (St. Louis, MO, USA).

### 2.9. Statistical Analysis

The collected data were subjected to rigorous statistical analysis employing the one-way analysis of variance (ANOVA) test, conducted using IBM SPSS software (version 20; SPSS Inc., Chicago, IL, USA). This analytical approach was employed to assess the treatment-induced variations in the hepatic fatty acid profile, lipid health indices, cholesterol levels, and tocopherol concentrations among the laying hens. For comparing means, Tukey's test was applied, and statistical significance was established at a threshold of *p* < 0.01. The outcomes are presented in the form of means ± standard error of the mean (SEM). Furthermore, to elucidate the interrelationship among the examined parameters, a principal component analysis (PCA) was conducted.

## 3. Results and Discussion

The use of hemp as a poultry feed ingredient is not common practice due to strict legislation on hemp cultivation, particularly in and outside European countries; however, in Morocco, in March 2021, the government passed Law No. 13–21 on the legal uses of cannabis, which authorizes the cultivation of cannabis for medical and industrial purposes, while prohibiting its use for recreational purposes.

Several studies have indicated that incorporating alpha-linolenic acid (ALA)-rich diets can strongly influence the fatty acid composition in the egg yolk and hen's liver, particularly in regards to ALA, EPA, and DHA [16, 32]. Research in the realm of incorporating hemp seed (HS) into the dietary regimen of laying hens predominantly centers on the utilization of whole hemp seeds, hemp oil, or other derivatives and products derived from the hemp plant [32–35]. Both birds and humans depend strongly on their liver for lipid metabolism [36]. Hepatic tissue occupies a central position in the orchestration of serum cholesterol and triglyceride levels through the modulation of their biosynthetic pathways and metabolic processes, as well as the intricate mechanisms governing the assembly, reutilization, and efflux of lipoproteins [37]. This present study demonstrates that the inclusion of HS at rates of 10%, 20%, and 30% in the diets of hens significantly raises levels of PUFAs n-3 in their liver when compared to the control group.

SFAs have been linked to an increased risk of developing heart disease. As a result, it is recommended to reduce their consumption. A diet enriched with HS impacted the concentration of SFAs (Table 3), composed mainly of palmitic acid (C16:0) and stearic acid (C18:0). When HS intake was raised from 0 to 30%, the level of SFAs in the liver fat reduced significantly (*p* < 0.01) from 38.6% to 36.9%. In agreement with these findings, Huang et al. [38] reported a noteworthy decrease in SFAs within the livers of hens that consumed 10% flaxseed. On the contrary, Juodka et al. [15] reported that there was no difference in the level of SFAs in the liver lipids of cannabis-cake fed ducks.

The total MUFAs in the liver are influenced by endogenous synthesis and/or intestinal absorption from the diet [39]. In the present study, the total MUFAs content was found to be significantly decreased from 29.6 to 22, as a result of a notable reduction of 0.2% in palmitoleic acid (C16:1) and oleic acid (C18:1) in the livers treated with (HS) diet, in comparison to the control group. Similar findings were also reported by Neijat et al. [16, 34], who demonstrated a significant reduction in hepatic MUFA levels at varying inclusion levels of HS (10%, 20%, and 30%). In addition, we observed a decrease in MUFA content in the egg yolk lipids when fed with HS [20]. These variations in MUFA levels can be attributed to a high level of omega-3 fatty acids, provided by cannabis seeds, which would reduce the expression of stearoyl-CoA desaturase SCD-1 in the liver, thereby decreasing oleic acid [40].

PUFA-n-3 fatty acids play crucial roles in numerous physiological activities, particularly in regulating the physical properties of cellular membranes, eicosanoid signaling, and gene expression of enzymes responsible for triglyceride storage [41]. However, it is noteworthy that the dietary requirements for animals with respect to these fatty acids are still not clearly defined [42]. It has been observed that the synthesis of omega-3 long-chain fatty acids is primarily regulated by substrate levels rather than gene expression [43].

Like many monogastric animals, laying hens exhibit a restricted endogenous enzymatic capacity to enzymatically alter the molecular structure of dietary fatty acids [44]. Nonetheless, they are capable of converting alpha-linolenic acid (ALA) to eicosapentaenoic (EPA) and docosahexaenoic (DHA) acids, as well as linoleic acid (LA) to arachidonic acid (ARA), by using specific elongase and desaturase enzymes [45]. The well-known competition between n-6 and PUFAs n-3 for their substrates, LA and ALA, respectively, by the same LC-PUFA-converting enzymes (desaturases and elongases) has been established [46]. Our results on PUFAs demonstrated a significant increase in the amount of PUFAs with the inclusion of HS, which is consistent with the recognized process of laying hens, converting ALA into long-chain metabolites [47]. A significant increase (*p* < 0.01) in DHA and ALA, and consequently PUFAs n-3, was observed in the livers of hens fed an HS-enriched diet, compared to the control group, which increased from 2.68%, 4.72%, 5.96% to 6.14%, respectively, for the control group, 10%, 20%, and 30% HS, respectively. However, for EPA, we found a nonsignificant increase when compared with control (*p* > 0.05). Some authors suggest that this may be due to the limited conversion of ALA to EPA in both animals and humans [48–50], or the subsequent conversion of EPA to eicosanoids to fulfill other functional requirements *in vivo* [51]. The HS supplementation also increased PUFAs n-6 levels in liver lipids from 29.04% for the control diet to 31.59%, 33.67%, and 34.7% for diets containing 10%, 20%, and 30% (HS), respectively. The enriched diets induced a significant rise in LA and ARA compared to the control diet, which is expected for two main reasons. First, the HS are abundant in LA, which is recognized as a substrate for PUFAs n-6 [45]. Second, there is co-occurrence of n-3 and PUFAs n-6 on the same PUFA conversion LC enzyme type. [46]. The competitive nature of n-3 and n-6 fatty acids for desaturation and elongation enzymes has been widely investigated in different experiments [52–54]. Most of these studies suggest that 18 : 3 n-3 is a preferential substrate for A6 desaturase to 18 : 2 n-6, given that an n-6/n-3 ratio of up to 3 is maintained [53, 55]. Consequently, the high concentration of ALA in Cannabis seeds can effectively promote the conversion of ALA to EPA and DHA over that of LA to ARA [56]. The marked increase in PUFA n3 relative to PUFA n-6 results in a substantial decrease in the n-6/n-3 ratio, which decreases as the level of HS inclusion increases until it reaches a plateau at 20% HS. These findings align with the majority of previously documented research outcomes [16, 34].

PUFAs and their ratios are important for regulating hypocholesterolemic indices. The PUFAs n-3 seem to play an essential role in the regulation of the thrombogenic index (TI), while the PUFAs n-6 are thought to have a major influence on the atherogenic index (AI). The consumption of healthy animal foods, such as meat products, should be characterized by low AI and TI values and high HDL-C indices [50]. According to other reports, the recommended levels for AI and TI should be below 1.0 [51]. The results of our study indicate that the hepatic lipid profile has a significant impact on lipid health indices. The AI was comparable in all treatments (0.6), except for HS-30% which showed a significant decrease compared to the control, while the TI was substantially lower in HS-10, HS-20, and HS-30 (0.85, 0.83, and 0.77, respectively) versus the control (1.02). We also found that the value of hepatic fatty acid saturation index decreased in hens subjected to the HS 10%, 20%, and 30% diets (22.65, 18.97, and 19.26, respectively) compared to the control diet (23.94). The value of the HDL-C/HDL-C + LDL-C (hH) indices obtained from the hens fed the HS diet was significantly higher than the value obtained from the control, which is beneficial for human health. Our finding is consistent with the results obtained by other authors based on the fatty acid profile of laying hens [23, 30, 57, 58]. According to the Food and Agriculture Organization (FAO), peroxidability index (PI), oxidative susceptibility (OS), desirable fatty acid (DFA), and nutritive value index (NVI) should be as high as possible and should be considered to have neutral or cholesterol-lowering effects [58, 59]. Our results show that the inclusion of HS in the diet of laying hens allows the increase of these different in dices compared to the control, and therefore the Cannabis seeds ecotype beldiya would allow to further improve the nutritional quality of laying hens (Table 4).

The liver is the primary site of cholesterol biosynthesis in laying hens, and the resulting cholesterol is incorporated into vitellogenin and/or very low-density lipoprotein particles that are secreted into the bloodstream and subsequently taken up by the oocytes [60]. In a previous study [61], we demonstrated that local ecotype hemp seed oil (HS) had an interesting effect on mice plasma lipid parameters, reducing the atherogenic index and LDL/HDL ratio but the hen's egg cholesterol content remained unaffected [20]. In the present study, we found that diets containing HS led to a reduction in cholesterol levels, which was significant at a level of *p* < 0.05 for the HS-10% diet, with levels of 94 and 82 mg/100 g for the control and HS-10% diet, respectively (Table 5). Although the observed decrease in cholesterol levels was small, it is likely due to a combination of factors related to the chemical composition of cannabis seeds (HS are particularly rich in phytosterols, protein, and insoluble fiber). Phytosterols mainly *β*-sitosterol have potential benefits in reducing hypercholesterolemia by blocking cholesterol absorption [62, 63]. They can also reduce cholesterol biosynthesis in the liver by modulating the activity of HMG-CoA reductase, thereby limiting the level of cholesterol formation [64]. Additionally, HS is rich in insoluble fiber and proteins. Plant proteins have been shown to have a cholesterol-lowering effect, which is largely due to the indigestible fraction that decreases intestinal cholesterol absorption, increases fecal sterol excretion, and improves the catabolism of lipoproteins containing the bulk of cholesterol by increasing the number or activity of LDL receptors [65]. Increasing fiber concentrations in diets reduces hepatic fat concentrations in laying hens by increasing fatty acid oxidation and decreasing fatty acid synthesis in the liver [66]. The viscosity produced by fiber is considered to be one of the key factors in reducing intestinal cholesterol absorption by binding directly to cholesterol, reducing its diffusion to the apical surface of the intestinal mucosa cells and being involved in the cholesterol emulsification in the intestinal lumen [67]. Concerning the liver weight, fat, and relative fat liver content, our findings show that the HS diet has no effect (*p* > 0.05) on these parameters.

Hemp seeds are acknowledged for their high levels of antioxidant constituents, including tocopherols, specifically *γ*-tocopherol [68, 69]. In the current study, there was a significant increase (*p* < 0.01) in total tocopherol levels, as well as *α*- and *γ*-tocopherol levels, only in the livers of chickens fed a diet containing a high amount of cannabis seeds (30-HS), than in the control group, while for the other treatments (10, 20-HS), there was an increase, compared to the control group, although not significant (*p* > 0.01). Skřivan et al. [17, 70] demonstrated that upon the inclusion of HS in the dietary regimen of hens, a substantial transference of *α*- and *γ*-tocopherol occurred in the egg yolk, liver, and muscle tissues and therefore enhanced their antioxidant potential. Korošec et al. [71] had reported that both isomers (*α*- and *γ*-tocopherol) played an important role in the expression of genes involved in inflammatory processes, immune response, and the metabolism of lipids and cholesterol.

**Figure 1:**
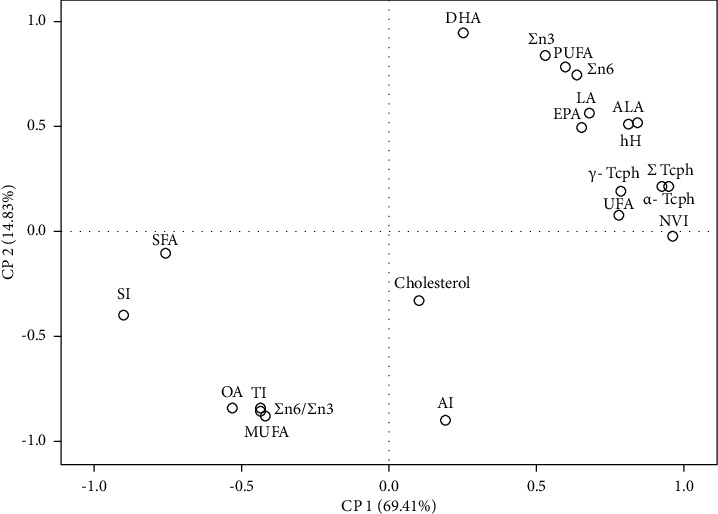
Correlation load of fatty acids, health indices, cholesterol, and tocopherol of the liver samples analyzed in the plane defined by 2 principal components.

**Figure 2:**
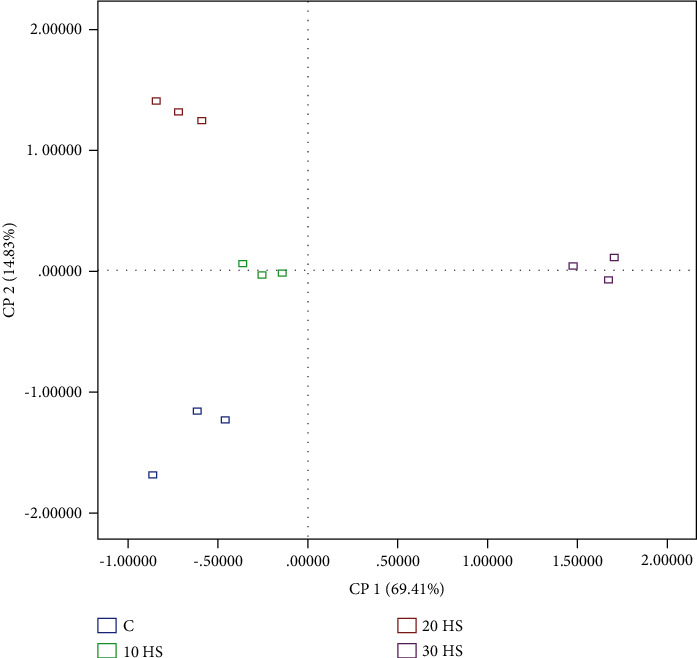
Dispersion of the four treatments (C, HS10, HS20, and HS30%) in the plane defined by 2 principal components.

To unravel the intricacies of the complex mixture comprising fatty acid classes, health lipid indices, tocopherols, and cholesterol, a principal component analysis (PCA) was conducted. The PCA model (depicted in Figures 1 and 2) revealed two significant components that accounted for 69.4% and 14.8% of the total variance of the original parameters (PC1 and PC2). Specifically, PC1 showed a positive correlation with tocopherols, NVI, and unsaturated fatty acids and a negative correlation with saturated fatty acids and SI, while PC2 revealed a positive correlation with DHA, polyunsaturated fatty acid and omega 3 fatty acid, and a negative correlation with cholesterol and AI.

Based on our data, we have observed a positive correlation between the concentration of ALA, EPA, DHA, and PUFA n-3 and some health indices. Furthermore, a robust correlation was discovered between tocopherols and long-chain fatty acid n-3. From the PCA conducted, we have identified four distinct groups, of which the control and 30-HS groups were the most prominent. The control group exhibited high values for SFAs, n-6/n-3 ratio, MUFAs, saturation index (SI), and thrombogenicity index (TI), while the 30-HS group showed high values for UFAs PUFA n-3, PUFA n-6, and tocopherols. The occurrence of these two distinct clusters therefore confirms the beneficial health effect of HS incorporation on the hepatic lipid profile.

## 4. Conclusion

The primary aim of our study was to assess the beneficial influence of incorporating nonindustrial Moroccan ecotype hemp seed (HS Beldiya) into the diet of laying hens on hepatic lipid metabolism. The results obtained in this study corroborate and extend our prior research on the productive performance and egg characteristics of laying hens. The results demonstrated that HS supplementation up to a level of 30% leads to advantageous changes in liver lipid composition, including a noteworthy increase in PUFAs n-3 levels, a decrease in cholesterol levels, and an increase in tocopherol levels. The most suitable concentration for the supplement appeared to be at a very low level, not exceeding 10% HS. Since the liver is the primary location of lipid metabolism, these modifications are expected to lead to favorable changes in plasma lipid parameters and may also improve the quality of meat produced. Consequently, future work may be conducted to investigate this hypothesis by analyzing the lipids of different muscles in laying hens fed nonindustrial Moroccan HS.

## Figures and Tables

**Table 1:** Formulation, proximate chemical composition, and nutrient profile of diets containing hempseed (HS) compared with the control.

Item	Diet			
	Control	HS-10	HS-20	HS-30
Ingredients (g·kg^−1^)				
Corn	588.29	462.14	380.01	212.94
Hemp seed	0	100	200	300
Soybean meal	255.85	104.59	95.63	110.94
Sunflower meal	16.85	130	130	130
Soybean oil	20.28	10	0	0
Calcium	86.09	73.79	89.34	89.27
DDGS^*∗*^	8.96	70	70	35.72
Di calcium phosphate	13.68	37.26	11.62	20
Sodium sulfate	0	2.37	3	2
Sodium chloride	1.83	1.12	2	2
DL-methionine	2.73	2.15	2.17	2
L-Lysine HCl	0.1	1.46	9.99	15
Vitamin premix^*∗∗*^	5	5	6.11	80
Mineral premix^*∗∗*^	0.4	0.12	0.13	0.13
Composition (calculated unless noted)				
AME (kcal/kg)^*∗∗∗*^	2990	3000	3000	3000
Crude fat (%)	5.06	7.14	8.48	11.10
Crude protein (%)	17.8	17.8	18	18
Calcium (g/kg)	39	40	40	40
Phosphorus available (%)	0.44	0.85	2.78	2.78
Sodium (g/kg)	1.1	2	2	2
*α*-Linolenic acid (%)	2.49	7.45	12.09	17.37
Lysine (g/kg)	9.35	8.64	8.71	8.74
Methionine (g/kg)	5.58	5.59	5.82	6.14
Threonine (g/kg)	6.90	6.63	6.94	6.91

^*∗*^DDGS: distiller's dried soluble grains; ^*∗∗∗*^AME available metabolizable energy. ^*∗∗*^Providing per g of premix, 11,00 IU vitamin A; 3,000 IU vitamin D3; 0.3 mg vitamin K3; 0.22 mg vitamin B1; 0.65 mg vitamin B2; 0.45 mg vitamin B6; 2 *μ*g vitamin B12; 1 mg pantothenic acid; 2.5 mg niacin; 0.4 mg folic acid; 15 IU vitamin E; 23 mg choline chloride; 0.30 mg sodium; 66 mg manganese; 80 mg iron; 70 mg zinc; 10 mg copper; 0.4 mg iodine; 36 mg magnesium; 0.10 mg selenium.

**Table 2:** Proximate and major fatty acid composition of hemp seeds (beldiya) utilized as a dietary constituent for laying hens.

Whole hemp seed (g·kg^−1^ DM)	
Dry matter	952.9
Ash	49.7
Crude protein	220.3
Crude fiber	275.4
Calculated metabolizable energy (kcal/kg)	3883.92
Lipid	330.2
Fatty acid (%)	
C16.0	7.68
C16.1 n-9	0.13
C18:0	3.03
C18:1 n-9	18.05
C18:1 n-7	1.05
LA	51.01
GLA	0.61
ALA	16.46
C20:0	0.79
C20:1 n-9	0.43
C24:0	0.27
Other FA	0.49
SFA	11.91
MUFA	19.72
PUFAs	68.38
UFA	88.09
PUFA n-3	16.53
PUFA n-6	51.85
PUFA n-6/PUFA n-3	3.14

LA, linoleic acid; ALA, *α*-linolenic acid; GLA, *γ*-linolenic acid; PUFAs, polyunsaturated fatty acids.

**Table 3:** Effect of dietary hemp seed on fatty acids profile (% total FA) in liver.

Fatty acids	Group				SEM	*p* value
	Control	HS-10	HS-20	HS-30		
C14:0	0.285^d^	0.208^c^	0.124^a^	0.152^b^	0.003	<0.001
C15:0	0.077^b^	0.066^a^	0.084^c^	0.114^d^	0.001	<0.001
C16:0	23.65^c^	22.45^b^	18.85^a^	19.11^a^	0.198	<0.001
C16:1	1.716^a^	1.178^b^	0.683^c^	0.735^c^	0.017	<0.001
C18:0	14.35^a^	14.32^a^	19.26^c^	17.44^b^	0.103	<0.001
C18:1	27.94^a^	25.31^c^	21.18^d^	21.46^d^	0.057	<0.001
C18:2n-6	22.60^a^	25.75^c^	24.84^d^	26.33^bc^	0.099	<0.001
C18:3n-6	0.099^a^	0.111^b^	0.143^c^	0.183^d^	0.001	<0.001
C18:3n-3	0.942^a^	1.905^c^	1.758^b^	2.528^d^	0.016	<0.001
C20:0	0.041	0.021	0.054	0.038	0.018	0.229
C21:0	0.181^b^	0.123^a^	0.12^a^	0.091^a^	0.011	<0.001
C20:3n-6	0.426^a^	0.438^a^	0.749^b^	0.683^b^	0.021	<0.001
C20:4n-6	5.919^b^	5.288^a^	7.936^c^	7.503^c^	0.102	<0.001
C20:3n-3	0.041^a^	0.049^a^	0.078^ab^	0.143^b^	0.017	<0.001
EPA	0.069	0.095	0.098	0.117	0.014	0.076
C24:0	0.034^b^	0.038^b^	0.016^a^	0.021^a^	0.002	<0.001
DHA	1.633^a^	2.671^b^	4.028^d^	3.353^c^	0.094	<0.001
SFA	38.62^c^	37.22^ab^	38.51^bc^	36.96^a^	0.277	0.001
UFA	61.38^a^	62.78^bc^	61.49^ab^	63.04^c^	0.277	0.001
MUFA	29.66^d^	26.47^c^	21.86^a^	22.21^b^	0.064	<0.001
PUFA	31.73^a^	36.31^b^	39.63^c^	40.84^c^	0.277	<0.001
n-3	2.684^a^	4.721^b^	5.962^c^	6.141^c^	0.109	<0.001
n-6	29.04^a^	31.59^b^	33.67^c^	34.70^d^	0.195	<0.001
n-6/n-3	10.88^a^	6.691^b^	5.648^b^	5.651^b^	0.364	<0.001

SFA, saturated fatty acids; UFA, unsaturated fatty acids MUFA, monounsaturated fatty acids; PUFA, polyunsaturated fatty acids; n-6: polyunsaturated fatty acids n-6; n-3: polyunsaturated fatty acids n-3; SEM, standard error of the mean; *p* significance. ^abcd^Mean marked with a different superscript letter within each row are significantly different.

**Table 4:** Effect of dietary hemp seed on health-related lipid indices of the liver of studied laying hens.

Items	Group				SEM	*p* value
	Control	HS-10	HS-20	HS-30		
SI	0.389^a^	0.361^b^	0.309^c^	0.305^c^	0.004	<0.001
DFA	75.73^a^	77.1^b^	80.75^c^	80.48^c^	0.21	<0.001
AI	0.638^a^	0.599^ab^	0.628^ab^	0.589^b^	0.030	0.001
TI	1.021^a^	0.853^bc^	0.834^bc^	0.781^c^	0.017	<0.001
OS	1150.8^a^	1386.9^b^	1329.9^c^	1478.3^d^	1.36	<0.001
hH	2.119^a^	2.313^b^	2.485^c^	2.579^c^	0.028	<0.001
HSFA	23.94^a^	22.65^b^	18.97^c^	19.26^c^	0.199	<0.001
NVI	1.788^a^	1.765^a^	2.145^b^	2.036^c^	0.014	<0.001

SI, saturation indices; DFA, desirable fatty acids; AI, atherogenicity indices; TI, thrombogenicity indices; OS, oxidative susceptibility; hH, hypo/hypercholesterolemic; HSFA, hypercholesterolemic saturated fatty acids; NVI, nutritive value indices; SEM, standard error of the mean, *p* significance. ^abcd^Mean marked with a different superscript letter within each row are significantly different.

**Table 5:** Effect of dietary hemp seed on weight, fat content, relative fat content, cholesterol, and tocopherol concentration of the liver of studied laying hens.

Items	Group				SEM	*p* value
	Control	HS-10	HS-20	HS-30		
Liver weight (g)	48.71	50.67	53.29	50.70	2.06	0.198
Fat liver (g/100 g)	5.09	4.92	4.74	4.84	0.22	0.438
Relative fat liver (%)	2.47	2.50	2.52	2.45	0.186	0.979
Cholesterol (mg/100 g liver)	95.14^b^	82.05^a^	90.39^b^	91.84^b^	3.062	0.013
*α*-Tocopherol (*μ*g/g liver)	8.055^a^	8.7651^a^	9.8885^a^	15.81^b^	0.912	<0.001
*γ*–Tocopherol (*μ*g/g liver)	0.936^ba^	0.651^a^	1.131^b^	1.661^c^	0.110	<0.001
Total tocopherol	8.991^a^	9.416^a^	11.02^a^	17.46^b^	0.549	<0.001

^abc^Mean marked with a different superscript letter within each row are significantly different.

## Data Availability

The data used to support the findings of this study are included within the article.
